# Effect of crocin and losartan on biochemical parameters and genes expression of FRMD3 and BMP7 in diabetic rats

**DOI:** 10.55730/1300-0144.5553

**Published:** 2022-09-13

**Authors:** Yaser MOHAMMADI, Mohammad ZANGOOEI, Fatemeh SALMANI, Azam REZAEI FARIMANI

**Affiliations:** 1 Qaen School of Nursing and Midwifery, Birjand University of Medical Sciences, Birjand, Iran; 2 Department of Clinical Biochemistry, School of Medicine, Birjand University of Medical Sciences, Birjand, Iran; 3 Departments of Epidemiology and Biostatistics, School of Health Social Determinants of Health Research Center, Birjand University of Medical Sciences, Birjand, Iran; 4 Cardiovascular Diseases Research Center, Birjand University of Medical Sciences, Birjand, Iran

**Keywords:** Diabetic nephropathy, biochemical parameters, crocin, losartan

## Abstract

**Background/aim:**

Diabetes is a multifactorial and growing disease, one of the severe complications of which is diabetic nephropathy (DN), which is the most common cause of chronic renal failure. FERM domain containing 3 (FRMD3) is responsible for maintaining the shape and integrity of nephron cells, and bone morphogenetic protein 7 (BMP7) helps maintain function and reduce kidney damage. This study aimed to evaluate the effect of crocin and losartan on biochemical parameters and the expression of FRMD3 and BMP7 genes in streptozotocin (STZ)-induced diabetic rats.

**Materials and methods:**

Forty male Wistar rats were randomly divided into five experimental groups as healthy, diabetic control (D), crocin, losartan, and diabetic rats treated with losartan-crocin (n = 8). A single dose of STZ (50 mg/kg intraperitoneally injection) was used to induce diabetes. Four weeks after induction of diabetes, rats received crocin (50 mg/kg) and losartan (25 mg/kg) daily for four weeks orally. Rats were sacrificed at the end of the intervention, and blood samples were taken to determine serum levels of glucose, urea, creatinine (Cr), malondialdehyde (MDA), and thiol. Real-time polymerase chain reaction (PCR) was used to assess the expression of the FRMD3 and BMP7 genes in the kidney samples.

**Results:**

Diabetes induction increased serum levels of glucose, Cr, urea, MDA, and thiol, but decreased BMP7 and FRMD3 genes expression. Treatment with crocin and losartan decreased these biochemical parameters and increased the expression of the BMP7 and FRMD3 genes.

**Conclusion:**

Crocin may be a promising therapeutic agent for preventing and improving diabetes-related kidney disease due to its antidiabetic and antioxidant properties.

## 1. Introduction

Today, the occurrence of diabetes mellitus (DM) is quickly increasing across the world [1]. Uncontrolled diabetes disrupts the function of multiple tissues and organs by triggering apoptosis, oxidative stress, and inflammation [2]. One of the most frequent consequences of diabetes is diabetic nephropathy (DN), characterized by renal dysfunction, vascular permeability in the glomerulus, and albuminuria [3]. The most main risk factors for the onset and development of DN are chronic hyperglycemia and hypertension, which are associated with the overproduction and accumulation of reactive oxygen species (ROS), and advanced glycation end products (AGEs), inflammatory factors, and gene expression alterations [4]. The FERM Domain Containing 3 (FRMD3) gene is one of the DN-related genes with the highest expression levels in renal proximal tubule cells [5]. It is responsible for maintaining the shape and integrity of nephron cells and belongs to the 4.1 cytoskeletal protein family [6]. FRMD3 transcript levels are significantly reduced with the development of DN. In a study, expression of FRMD3 in diabetic mice was shown to be lower than in nondiabetic [7]. Furthermore, single nucleotide polymorphisms (SNPs) in the FRMD 3 gene, particularly the SNP rs1888747 in the promoter region, have been linked to the initiation and progression of DN [8]. According to research, this SNP influences the bone morphogenic protein (BMP) pathway [9]. These data suggest that FRMD3 and several BMP pathways are interconnected. The epithelial cells of the urethra and distal tubules express BMP7 endogenously [10]. Its expression rises in the early stages of DN to help repair the damage, but it declines as the disease develops [11]. As a result of its antifibrotic action, BMP7 may be useful in preventing the advancement of DN [6]. Because the pathogenic mechanism causing DN is unknown, and effective therapy options are limited, novel DN management strategies are required. Angiotensin II receptor blockers (ARBs) and angiotensin-converting enzyme (ACE) inhibitors are the most widely used drugs to prevent and improve DN, however they also cause adverse effects. Consequently, scientists have focused their efforts on herbal remedies with fewer side effects. One of these natural compounds is crocin. Crocin is one of the effective ingredients of the saffron plant. It has a hypoglycemic impact and promotes pancreatic beta-cell activity [12,13]. Crocin also has antiinflammatory, antioxidant, and antitumor effects [14–16]. Based on the antidiabetic properties of crocin, this study aimed to evaluate the effect of crocin and losartan on biochemical parameters and the expression of FRMD3 and BMP7 genes in streptozotocin (STZ)-induced diabetic rats.

## 2. Materials and methods

### 2.1. Chemicals and drugs

STZ and crocin were purchased from Sigma-Aldrich (St Louis, MO, USA). Losartan was purchased from ACTOVERCO pharmaceutical factory (Karaj-Iran). Losartan and crocin were dissolved in distilled water and administered orally.

### 2.2. Animals

Forty male Wistar rats, weighing 200–250 g, bought from the Experimental Research Center of Birjand University of Medical Sciences. The study was conducted under ethical considerations in animal studies. The ethics committee of Birjand University of Medical Sciences approved it (IR.BUMS.REC.1399.301). Rats were housed for a week in standard cages in a room with regular light cycles (12–12 h light/dark), a temperature of 20–26 °C, a humidity of 60%–80%, and free access to water and food (standard rat chow).

### 2.3. Experimental design

Eight rats were randomly selected as a healthy control group, while the remaining 32 rats became diabetic with STZ. Diabetes was induced by injecting a single dose of STZ (50 mg/kg) intraperitoneally as explained by Elsherbiny et al. [17]. To avoid hypoglycemia, rats were given free access to a 15% glucose solution following STZ injection. Fasting blood sugar (FBS) was measured from the tail vein using a standard glucometer Accu-Check (Roche, Germany) 72h after STZ injection. Rats with FBS > 250 mg/dL were confirmed as diabetic. Four weeks after STZ injection, diabetic rats were randomly divided into four groups (n = 8 in each group) using a block size of 4. These groups included diabetic control group, diabetic groups treated with crocin (50 mg/kg), losartan (25 mg/kg), and losartan-crocin (25 + 50 mg/kg). The treatments were done by gavage daily for four weeks.

### 2.4. Sample collection

At the end of the period (8 weeks), rats were anesthetized with an intraperitoneally injection of ketamine (100 mg/kg) and xylazine (10 mg/kg). Blood samples were drawn from each rat’s inferior vena cava. To extract the serum, the samples were centrifuged at 3000 revolutions per min (RPM) for 10 min, then stored at −20 °C until used in biochemical and oxidative stress assays. Both kidneys were removed, washed in cold saline, and weighed for the kidney/body weight index calculation. They were immediately frozen in liquid nitrogen and transferred to −80 °C for future analysis. Also, for 24-h urine collection, one day before to sacrifice, rats were put in metabolic cages.

### 2.5. Measurement of biochemical parameters

Serum levels of glucose, urea, creatinine (Cr), and urine total protein (UTP) were measured using standard kits (produced from Pars Azmoun, Iran) using an automatic device (Prestige 24i, Japan).

### 2.6. Measurement of malondialdehyde (MDA)

The serum MDA levels were measured using the Thiobarbituric Acid Reactive Substances (TBARS) method (Zantox kit-Iran). This assay is based on the formation of a pink chromogenic when MDA reacts with TBA. A fluorometer was used to measure the MDA-TBA adduct (Ex/Em = 515/553 nm).

### 2.7. Measurement of thiol

A sulfhydryl group (also known as a “thiol group”) is ubiquitous in our body and mostly found in the oxidized form as disulfide linkages. Thiol was measured using commercial kits (Zantox-Iran). Ellman’s reagent (5, 5′-dithiobis-(2-nitrobenzoic acid); DTNB), which releases 2-nitro-5-thiobenzoate (NTB) when reacting with free thiol, is used to quantify free thiol. NTB has a bright yellow color that can be measured with a spectrophotometer between 410 and 415 nm and an extinction coefficient of 14,150/M-cm.

### 2.8. Total RNA extraction and real-time polymerase chain reaction (real-time PCR)

Frozen kidney tissues (50 mg) were homogenized in liquid nitrogen using a pestle. Total RNA extracted using kiazol Reagent (KIAZIST, Iran) according to the manufacturer’s instructions. The quality and integrity of isolated RNA were assessed using a 1% agarose gel electrophoresis. A Nano Drop Ultraviolet (UV) spectrophotometer (Biotech-America) was used to determine the RNA concentration and purity. Following the manufacturer’s protocol, the qualified RNA is converted to cDNA using a reverse transcriptase reaction with a cDNA synthesis kit (Pars Tous, Iran) and analyzed using the 2X SYBR Green real-time PCR master mix (Pars Tous, Iran) on a Step One real-time PCR detection system (Applied Bio systems, USA). One cycle at 95 °C for 10 min was followed by 40 cycles at 95 °C for 15s, 65 °C for 30 s, and 72 °C for 30 s in the amplification protocol. β-actin gene was used as an internal control gene.

The delta cycle threshold (ΔCt) method was used to detect transcript levels. All the RT-PCR reactions were run in duplicate. A sample was placed in each run from each group. FRMD3 and BMP7 genes with β-actin internal control gene were examined in each run. The sequence of primers is reported in Table 1.

### 2.9. Statistical analysis

All data from the experimental groups were presented as means ± standard error of mean (SEM). The Shapiro-Wilk test was used to test the normality hypothesis and in the case of hypothesis, analysis of variance and appropriate post hoc tests were used to compare the groups. If the normality hypothesis is not established, the nonparametric equivalent of the tests (Wilcoxon-Kruskal-Wallis) was used. P < 0.05 was considered to be statistically significant. The analysis was performed using IBM SPSS Statistics 16.0 software (SPSS, Inc., Chicago, IL).

## 3. Results

### 3.1. The effect of crocin and losartan on FBS levels

**Figure 1:**
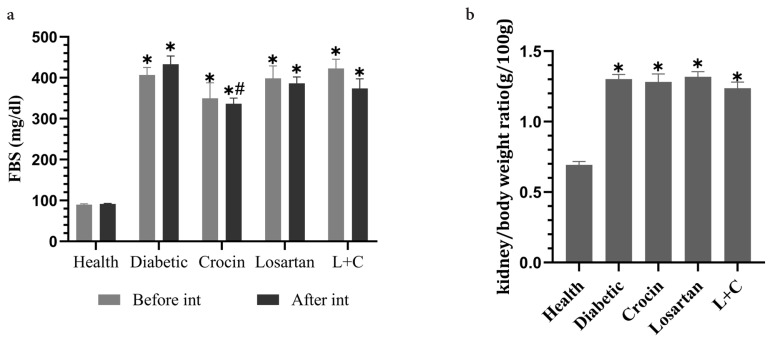
The effect of crocin and losartan on **a**: FBS, **b**: kidney/body weight ratio. Data were represented as mean ± SE. L+C: Losartan-crocin, N = 8. *p < 0.05: Significant difference with health group. #p < 0.05: Significant difference with diabetic group.

Figure 1a, depicts FBS concentrations before and after the intervention. Before the intervention, diabetic rats’ FBS levels were found to be significantly higher than healthy rats (p = 0.001). The FBS levels in the crocin group decreased significantly after the intervention as compared to the diabetes group (p = 0.001). In the losartan and losartan-crocin treatment groups, FBS levels decreased compared to the diabetic control group, but these changes were not significant. Also, in the intragroup comparison before and after the intervention, FBS levels increased in the diabetic group and decreased in the treated groups, but these changes were not significant.

### 3.2. The effect of crocin and losartan on kidney/body weight ratio

The kidney/body weight ratio is shown in Figure 1b. The kidney/body weight ratio in the diabetic group showed a significant increase compared to the healthy group (p = 0.001). The crocin and losartan-treated groups had a lower ratio than the diabetic group, but the differences were not significant.

### 3.3. The effect of crocin and losartan on UTP levels

**Figure 2:**
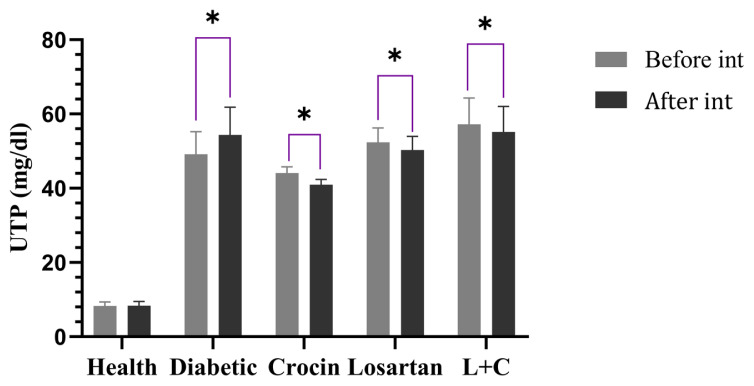
The effect of crocin and losartan on UTP. UTP: Urine total protein. Data were represented as mean ± SE. L+C: Losartan-crocin, N = 8. *p < 0.05

Figure 2 depicts the changes in UTP. Before the intervention, all diabetic rats had significantly higher UTP levels than healthy rats (p = 0.001). UTP levels decreased after the intervention in the crocin and losartan groups compared to the diabetic group, but these changes were not significant. Also, in the intragroup comparison before and after the intervention, UTP levels increased significantly in the diabetic group (p = 0.02) and decreased in the crocin (p = 0.001), losartan (p = 0.001), and losartan-crocin groups (p = 0.001).

### 3.4. The effect of crocin and losartan on serum levels of urea and Cr

Table 2 shows the serum levels of urea and Cr. The diabetic group had significantly higher serum urea levels than the healthy group (p = 0.001). Also, serum urea levels in the crocin-treated group were significantly lower compared to the diabetic group (p = 0.001). Serum Cr levels also increased in the diabetic group compared to the healthy group and decreased in the treated groups compared to the diabetic group, but these changes were not significant.

### 3.5. The effect of crocin and losartan on serum levels of thiol and MDA

**Figure 3:**
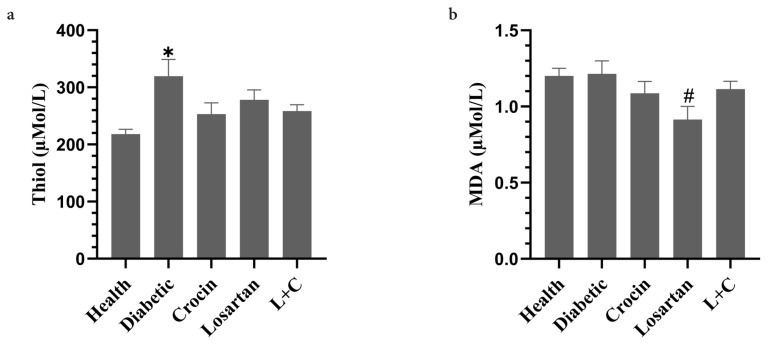
The effect of crocin and losartan on serum levels of **a**: Thiol, **b**: MDA. Data were represented as mean ± SE. L+C: Losartan-crocin, N = 8. *p < 0.05: Significant difference with health group. #p < 0.05: Significant difference with diabetic group.

Figure 3a depicts serum levels of thiol. Serum levels of thiol in the diabetic group were significantly higher compared to the healthy group (p = 0.004). In other groups, the serum levels of the thiol were lower than the diabetic group, but these changes were not significant.

Figure 3b depicts serum levels of MDA. The diabetic group’s MDA levels were higher than the healthy groups, but the difference was not significant. The losartan group had a significantly decrease serum MDA levels than the diabetes group (p = 0.04). The groups of crocin and losartan-crocin had lower serum MDA levels compared to the diabetic group, but these changes were not significant.

### 3.6. The effect of crocin and losartan on the relative expression of BMP7 and FRMD3

The relative expression of BMP7 and FRMD3 genes is shown in Table 3. The results showed that the relative expression of BMP7 and FRMD3 in the diabetic group significantly decreased compared to the healthy group (p = 0.03, p = 0.001, respectively). In the crocin and losartan-treated groups, the relative expression of the desired genes increased compared to the diabetic group, but these changes were not significant.

## 4. Discussion

DN is a chronic kidney disease that affects patients who have had diabetes for a long time. STZ was employed to induce type 1 diabetes in the current investigation. In the present study, diabetic rats had significantly higher serum levels of FBS, urea, Cr, MDA, and thiol, as well as a higher kidney/body weight ratio than healthy rats. The levels of FRMD3 and BMP7 gene expression were significantly lower in diabetic rats than in healthy rats. Crocin was able to ameliorate the adverse metabolic consequences in diabetic rats.

We found that crocin significantly reduced FBS in diabetic rats, which was in line with previous findings. According to several studies, crocin significantly increased peripheral insulin sensitivity and glucose uptake by phosphorylating acetyl-CoA carboxylase (AMPK/ACC), mitogen-activated protein kinases (MAPK), transferring glucose transporter-4 (GLUT-4) to cell membranes, and reducing oxidative stress [18–20]. In diabetic rats given losartan, FBS levels were also lowered. Losartan improves insulin sensitivity and glucose uptake [21].

Increased kidney/body weight ratio is one of the manifestations of DN. When compared to the healthy group, this ratio increased significantly in the diabetes group. Hyperglycemia can lead to an increase in renal hyperplasia by increasing the production of various growth factors and their receptors, also increased renal protein excretion, extracellular matrix accumulation (ECM), and mesangial matrix enlargement [22]. The crocin-treated group had a decreased kidney/body weight ratio compared to the diabetic control group, but these changes were not significant. It is suggested that probably by increasing the dose of crocin and the duration of the intervention, a significant effect on the ratio of kidney weight to body weight may be observed. Proteinuria is one of the symptoms of DN, which shows kidney damage caused by hyperglycemia and hypertension [23]. The results of this study revealed that UTP levels were considerably higher in diabetic group than in healthy group. UTP levels significantly decreased in the crocin-treated group. Crocin reduced proteinuria by lowering serum glucose and oxidative stress, according to Hadeer et al. [14]. UTP also decreased in losartan-treated diabetic rats. Lee et al. showed that treatment with losartan for 3 and 24 months reduced proteinuria by 30% and 43%, respectively [24].

With the progression of diabetes and increased levels of free radicals, it leads to damage to the kidney glomeruli, which the kidney’s ability to maintain a constant filtration rate to remove Cr and other nitrogenous components from the blood deteriorates, resulting in higher serum urea and Cr levels [25]. Crocin was able to significantly reduce urea levels compared to the diabetic group. It seems that this beneficial effect of crocin is due to its antioxidant properties. The study of Yaribeygi et al. supports our study [3]. Other treatment groups reduced serum levels of urea and Cr, but this decrease was not statistically significant. Our results show that by increasing the dose of crocin and losartan, high levels of urea and Cr will approach the level of the healthy control group.

Oxidative stress is a crucial factor in DM and its complications [26]. By altering normal glucose homeostasis, free radicals can cause DM and insulin resistance in peripheral organs [27]. According to our findings, in rats treated with crocin and losartan-crocin, serum levels of MDA and thiol decreased compared to the diabetic control group and approached the healthy group, but this decrease was not statistically significant. Crocin has been shown to decrease oxidative stress both directly and indirectly by binding to free radicals and activating antioxidant enzymes [28]. Crocin binds directly to the superoxide anion and blocks its harmful effects, according to a study by Pham et al. [29]. In this study, losartan significantly reduced serum MDA levels compared to the diabetic group. Ang II is activated by hyperglycemia. The principal effects of Ang II are the activation of nicotinamide adenine dinucleotide phosphate (NADPH) oxidase and the production of ROS [30]. Losartan may decrease oxidative stress by blocking the Ang II type 1 receptor and lowering the activity of NADPH oxidase [31].

The FRMD3 gene is a candidate gene in type 1 DN and is involved in the pathophysiology of the disease. It has a FERM domain, which interacts with transmembrane proteins and actin to maintain cell integrity. FRMD3 is expressed differently in different parts of the rat kidney, with the glomerulus expressing the most [7,32]. Because FRMD3 is involved in nephron function, its expression appears to be reduced in DN. FRMD3 transcript levels are significantly reduced with the development of DN and its expression was found to be lower in rats with DN compare to nondiabetic rats [7]. A study found that FRMD3 expression was suppressed at the RNA level in the kidneys of diabetic patients [33]. According to our findings, the renal expression of FRMD3 was significantly lower in the diabetic group compared to the healthy group. While the relative expression of FRMD3 increased in the crocin and losartan-treated groups compared to the diabetic group, but the difference was not statistically significant.

Our results were consistent with the study of Pezzolesi et al. [5]. They reported that FRMD3 expression was increased in human proximal tubular cells. As a result, FRMD3 expression data indicate that this gene is associated with the early development of DN. In addition, FRMD3 is predicted to suggest alternative pathways for the role in DN through interaction with other genes. Recently, FRMD3 has been shown to bind to multiple bone morphogenetic protein (BMP) members via the transcription factor binding site (TFBS) [8, 33]. However, the mechanism mediating the binding between members of the FRMD3 and BMP pathways is still unknown, and no data at the protein, RNA, or microRNA level are available to elucidate this association [7]. Because the link between the FRMD3 gene and diabetic nephropathy has recently been identified, very few studies are available focusing on mRNA and protein expression.

BMP7, a renal tubular growth morphogen, has recently been shown to help maintain renal function and reduce kidney damage [34]. The findings of our study showed that the relative expression of BMP7 in the diabetic group was significantly decreased compared to the healthy group. One of the main reasons for decreased BMP7 expression in diabetic rats is glucose-induced ROS overproduction [35,36]. Yang et al. reported a transient rise in renal BMP7 in rats during the primary stages of diabetes, but the level of BMP7 in the diabetic kidney decreased as diabetes progressed, compared to the healthy control group [37]. BMP7 helps podocytes keep their shape and integrity and reduces tubular cell apoptosis and protects renal function by having antifibrogenic properties [38,39]. The antifibrogenic activity of BMP7 may be mediated by inhibition of TGFβ signaling [40].

We found that renal expression of BMP7 increased after 4 weeks of treatment with crocin and losartan, but the changes were not statistically significant. Because high glucose-induced oxidative stress is linked to lower BMP7 expression, crocin and losartan may increase BMP7 expression by lowering ROS levels. Previous studies have shown that crocin can reduce free radical damage with its glucose-lowering and antioxidant effects [41]. As a result, crocin and losartan can reduce kidney damage and improve kidney function by reducing ROS and increasing endogenous BMP7 expression.

## 5. Conclusion

The finding of the present study showed that crocin can improve renal function indicators and the expression level of genes associated with DN (FRMD3 and BMP7) with its antioxidant and antidiabetic properties. In addition, our results showed that crocin in combination with losartan increases the effectiveness of losartan. Consequently, we suggest that crocin may be a potential therapeutic agent for diabetes and its side effects in combination with chemical drugs. However, more human studies are needed to reach definitive findings.

## Figures and Tables

**Table 1:** Primer sequences for real-time PCR.

Gene	Primer sequence
β-actin	*Forward*: 5′-CGCGAGTACAACCTTCTTGC-3′ *Reverse*: 5′-GTCTACAACATGATCTGGGTCA3′
BMP7	*Forward:* 5′-GCTTCGTCAACCTAGTGGAG-3′ *Reverse*: 5′-CCTTATAGATCCTGAACTCGGC-3′
FRMD3	*Forward*: 5′-CTCACCCATGCAAGGATTCA-3′ *Reverse*: 5′-AATACAACAAAACCCGCAGC-3′

**Table 2:** The effect of crocin and losartan on serum levels of urea and Cr.

Variable	Groups				
	Health	Diabetic	Crocin	Losartan	L+C
Urea (mg/dL)	39.37 ± 1.49	143 ± 11.5*	100.25 ± 5.38*#	138.57 ± 9.22*	114.71 ± 4.44*
Cr (mg/dL)	0.68 ± 0.06	0.85 ± 0.02	0.77 ± 0.07	0.82 ± 0.01	0.8 ± 0.03

Data were represented as mean ± SE. L+C: Losartan-crocin, N = 8.

* p < 0.05: Significant difference with health group.

# p < 0.05: Significant difference with diabetic group.

**Table 3:** Comparison of mean ΔCt of BMP7 and FRMD3 genes in the studied groups.

Variable	Groups				
	Health	Diabetic	Crocin	Losartan	L+C
BMP7	1.46 ± 0.27	3.39 ± 0.67*	2.45 ± 0.35	2.74 ± 0.44	2.66 ± 0.81
FRMD3	10.29 ± 0.43	14.97 ± 0.86*	13.42 ± 0.58*	14.47 ± 0.49*	14.26 ± 0.32*

Data were represented as mean ± SE. L+C: Losartan-crocin, N = 8.

* p < 0.05: Significant difference with health group.
